# Effect of skin dielectric properties on the read range of epidermal ultra-high frequency radio-frequency identification tags

**DOI:** 10.1049/htl.2016.0072

**Published:** 2017-04-24

**Authors:** Dumtoochukwu O. Oyeka, John C. Batchelor, Ali Mohamad Ziai

**Affiliations:** School of Engineering and Digital Arts, University of Kent, Canterbury, Kent, CT2 7NT, UK

**Keywords:** skin, permittivity, electrical conductivity, bioelectric phenomena, radiofrequency identification, biomedical measurement, skin dielectric properties, epidermal ultrahigh frequency RFID tags, human tissue conductivity, permittivity, body mass index values, radiofrequency identification tags

## Abstract

This Letter presents an investigation of the effect of human tissue conductivity and permittivity on the performance of epidermal transfer tattoo ultra-high frequency radio-frequency identification (RFID) tags. The measurements were carried out on 20 individuals and the variations in the measured dielectric properties correlate well with variations in the measured tag read range on the individuals and to a lesser extent with their body mass index values. Simulation results also showed the effects of permittivity and conductivity on the designed resonance frequency of the RFID tag.

## 1 Introduction

There has been an increase in the use of body mounted devices such as radio-frequency identification (RFID) tags and other sensors in recent years. These devices can be used in hospital environments for patient monitoring and tracking [1] and also as sensors [2] due to their non-invasive nature. As these devices get more miniaturised and ever closer to the body, it is important to understand their interaction with the human body which is known to have an adverse effect on the wireless performance of electromagnetic devices in close proximity as have been acknowledged in [3, 4]. The influence of the electrical properties of the skin and different body types on the functioning of epidermal UHF RFID tattoo tags of the kind reported in [5–8] is studied in this Letter. This stems from the differences in tag read ranges observed when an identical tag is measured on different individuals.

The electrical properties of the human body vary on an individual basis [9], making it in inevitable that transfer tattoo tags which are less than 26 µm from the skin will have performance dependent on the specific person on which they are mounted. The electrical properties of the body are affected by factors such as the skin thickness which is influenced by environment [10] and ethnicity [11]. Also the density of the underlying fat tissues, muscles and bone in the area being considered can affect the electrical properties of the body [12, 13] studied the influence of general lossy dielectric objects on the resonance frequency of RFID antennas. The aim of this Letter is to establish directly the degree of influence skin permittivity and conductivity variations have on the performance of the transfer tattoo tag in terms of read range when mounted on the forearm.

## 2 Experimental setup

To measure the dielectric properties of the human body, a probe based dielectric system by Schmid & Partner Engineering AG [14] was used. This is a high precision dielectric parameter (permittivity, loss tangent and conductivity) measurement device. It can be used in the electronics, food, medical and chemical industries for the measurement of solids, semi-solids and liquids for frequencies from 200 MHz to 20 GHz. The dielectric properties of the materials under test are calculated from the reflection coefficient at the probe-material interface with the method presented in [15]. It should be noted that the reflection coefficient measured by the probe is composed of multiple reflections from the tissues underneath the skin and hence the presented results are influenced by subdermal layers. This is because of the penetration of the electric field of the probe into the underlying deeper tissues [16].

Twenty subjects participated in the study with ages ranging from 21 to 55. These individuals were chosen to represent diverse body types. Physical observation showed them to have different levels of fat and muscle density and this meant that the circumferences of their arms were different. The body mass index (BMI) was calculated for all individuals from:

(1) $${\rm BMI} = \displaystyle{{{\rm Body}\, \, {\rm weight}\, \, \lpar {\rm kg}\rpar } \over {{\lpar {\rm Height}\, \, \lpar {\rm m}\rpar \rpar }^2}}\eqno\lpar 1\rpar $$

**Fig. 1:**
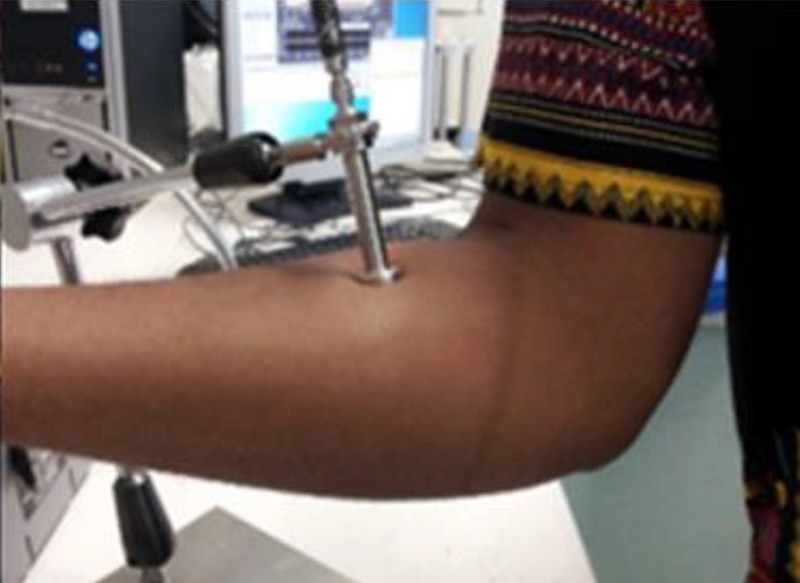
Measurement of body dielectric parameters

The tag placement and position of the probe was in the same location on the arm for all individuals and it was ensured that the probe aperture was flat on the skin in order to eliminate the possibility of air gaps, as shown in Fig. 1 [17].

## 3 Measurement results

**Fig. 2:**
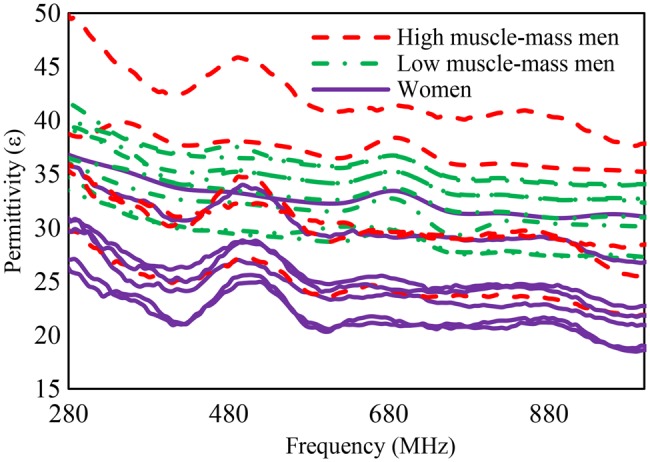
Measured skin permittivity of 20 individuals

**Fig. 3:**
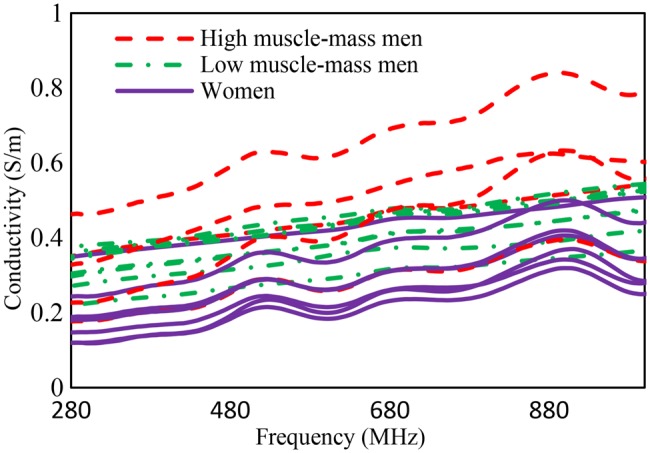
Measured skin conductivity of 20 individuals

The measured results sorted for BMI are presented in Table 1 while the measured relative permittivity and conductivity plots are shown in Figs. 2 and 3, respectively.

**Table 1:** Measured dielectric values sorted by participant BMI

Subject type	BMI, kg/m^2^	Relative permittivity, *ε*_r_	Conductivity, S/m	Arm circumference, cm
woman	17.10	21.56	0.32	21.00
woman	18.10	25.36	0.41	22.00
woman	18.40	22.77	0.38	23.00
woman	20.60	23.32	0.35	24.20
man (LM)	21.70	34.39	0.51	25.50
woman	24.50	25.54	0.43	24.50
man (LM)	24.60	32.85	0.49	24.00
woman	25.00	31.12	0.48	25.00
woman	25.10	30.49	0.51	25.30
man (LM)	26.50	34.23	0.49	26.50
man (HM)	26.70	31.14	0.64	27.00
man (LM)	27.10	32.80	0.49	26.50
man (HM)	27.20	40.96	0.84	29.00
man (HM)	27.40	30.44	0.62	29.00
man (LM)	27.60	29.44	0.38	25.50
man (HM)	27.80	24.61	0.40	28.00
man (LM)	29.40	30.49	0.44	24.50
man (HM)	30.40	35.76	0.51	32.00
man (LM)	31.70	31.17	0.44	32.50
man (LM)	33.80	27.52	0.39	31.00

LM: low muscle-mass, HM: high muscle-mass.

The measurements show that the individuals with higher fat content on their arms had lower permittivity and conductivity values than the other participants. In contrast, individuals with higher muscle density in the arms compared to fatty tissues had higher permittivity and conductivity values. The presented arm circumferences and BMI in Table 1 indicate the physical differences between the individuals.

**Fig. 4:**
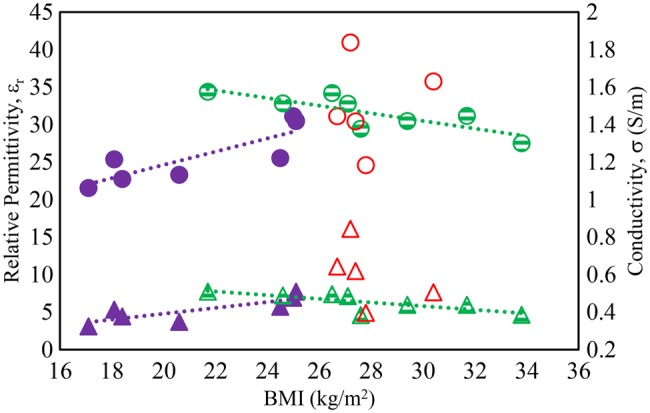
Effect of BMI on body dielectric properties at 867 MHz. Circles: ε_r_, Triangles: σ. Solid: women, strip: men (LM), hollow: men (HM)

Fig. 4 shows the relationship between the BMI and the measured dielectric properties. BMI does not distinguish between fat and muscle-mass and five male subjects were observed to have a high muscle-mass (HM) and low fat content compared to the other participants. While the female subjects and males with low muscle-mass (LM) showed different trends between BMI and permittivity/conductivity, the data for males with HM was too scattered to plot a trend. The permittivity and conductivity data for the women and men with LM have correlation (*r*) values greater than 0.7, which indicates acceptable trends for biological values (where 0 < |*r*| < 1 represents zero to perfect data correlation with the trend line).

**Fig. 5:**
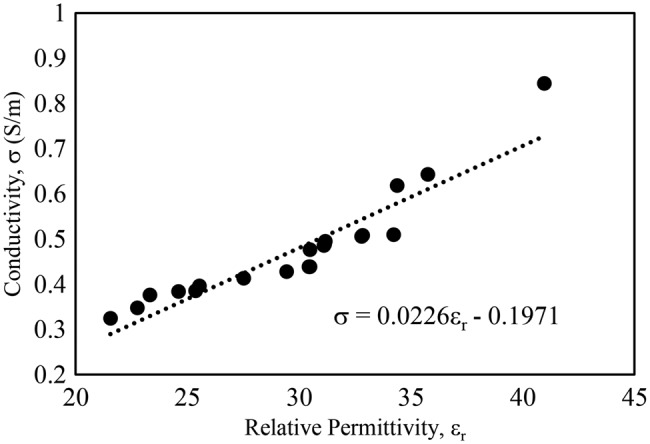
Relationship between measured relative permittivity and conductivity at 867 MHz

Fig. 5 shows the plot of the relationship between conductivity and relative permittivity. There is a near linear relationship with *r* = 0.93 between the measured relative permittivity and the conductivity of the individuals at 867 MHz. From this plot, an approximate value of permittivity can be found for a given conductivity and vice versa. This is useful for modelling human phantoms for epidermal tag design.

**Fig. 6:**
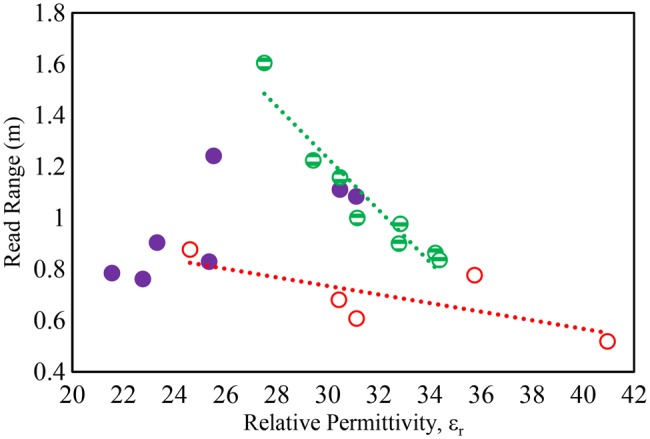
Detuning effect on tag read range due to tissue permittivity at 867 MHz) Solid symbol: women, strip: men (LM), hollow: men (HM)

**Fig. 7:**
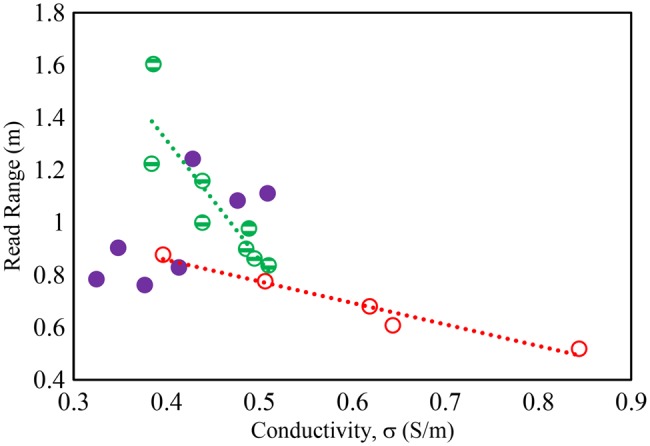
Detuning effect on tag read range due to tissue conductivity at 867 MHz. Solid symbol: women, strip: men (LM), hollow: men (HM)

To assess the tissue effect on epidermal RFID, the tag introduced in [8] was mounted on the arm of the individuals and the tag read range was measured using the Voyantic Tagformance measurement kit [18]. The measured read ranges were related to the measured dielectric properties as illustrated in Figs. 6 and 7. The measurements indicate that both permittivity and conductivity of the arm tissue correlate strongly with the achieved read range for a skin mounted tag on men with LM, and reasonably well for men with HM. However, there is a poor correlation between read range and permittivity, and conductivity for the women. Applying the tag to the different individuals can cause read range to fall to a third of the maximum value with the lower values being observed on the muscled men.

**Fig. 8:**
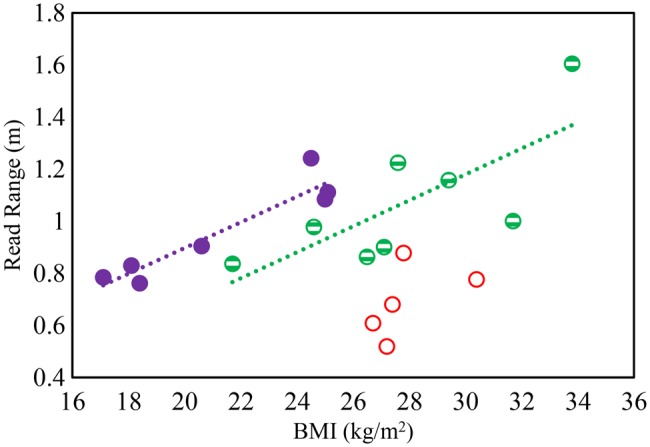
Detuning effect of BMI (kg/m^2^) on tag read range at 867 MHz. Solid symbol: women, strip: men (LM), hollow: men (HM)

Fig. 8 shows the measured read range plotted against BMI for the test population and it is clear that there are different trends for the body types, though the correlations are reasonable for women and LM men, though there is a significant spread about the trend line for the men. There is no significant trend for the men with HM to relate read range to BMI. However, the trend lines for women and LM men give indications of the read ranges likely to be obtainable for skin tag applied to those body types. Table 2 shows the correlation factor *r* for the data in Figs. 4 and 6–8. It is clear that BMI allows reasonable prediction of skin permittivity and conductivity for the women and men with LM in their arms. BMI also allows good prediction of tattoo tag read range for these body types, though there is a spread around the trend line for the men. However, men with HM have a significant scatter for BMI related to permittivity, conductivity and read range. For this body type, read range is reasonably well correlated to skin permittivity and conductivity.

**Table 2:** Correlation factor *r* for BMI related to skin permittivity, conductivity, and tattoo tag read range, and for read range related to skin permittivity and conductivity

	Correlation, *r*				
	BMI/*ε*_r_	BMI/*σ*	Read range/*ε*_r_	Read range/*σ*	BMI/read range
women (BMI < 31.2)	0.84	0.82	0.68	0.71	0.94
men (LM)	−0.82	−0.76	−0.95	−0.88	0.75
men (HM)	0.16	−0.46	−0.73	−0.98	0.51

A factor in the change of read range observed for the different individuals is the shift in tag resonance frequency due to dielectric loading. RFID tags are usually designed at overly high frequencies when in free space so they will become resonant at the desired band when they are loaded by the mounting platform. However to compensate for the variable tissue permittivities, sufficiently wide band RFID tag designs are recommended so that an acceptable read range can be obtained when there is a variable shift in the designed resonance frequency.

**Fig. 9:**
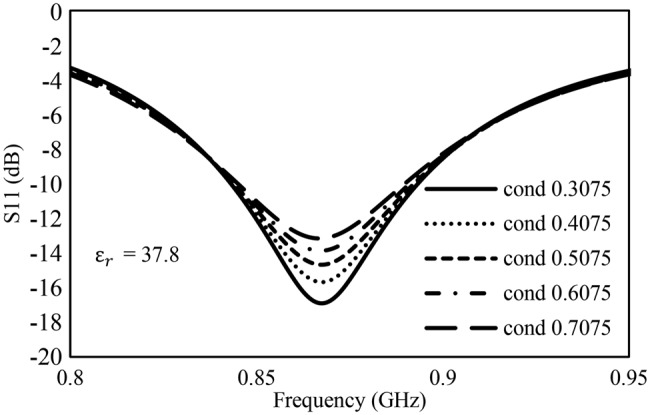
Effect of conductivity (S/m) on transfer tattoo tag input reflection coefficient, S_11_

**Fig. 10:**
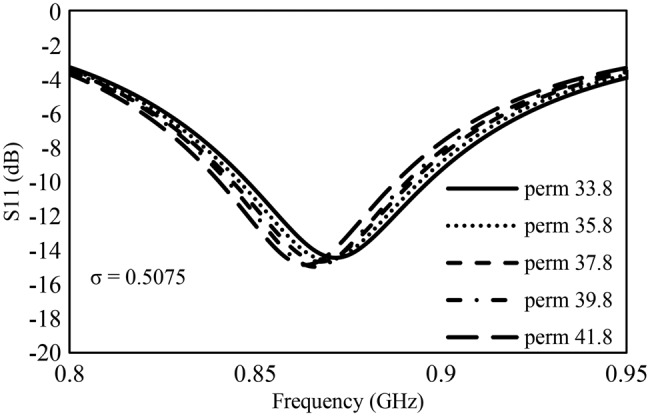
Effect of relative permittivity on transfer tattoo tag input reflection coefficient, S_11_

To better understand the effects of the skin dielectric parameters on the tag resonance frequency, an electromagnetic simulation of the tag on a skin model was carried out using the measured values of permittivity and conductivity in the CST microwave studio ® program. The *S*_11_ (tag input reflection coefficient) results are shown in Figs. 9 and 10. In these plots, the position of the *S*_11_ null indicates the tag is accepting energy at these frequencies. A deeper null means that the tag accepts a greater amount of input power and will have a bigger read range.

From Fig. 9, it can be seen that variation in the conductivity of the skin does not lead to change in frequency but rather affects the match of the antenna with the chip. This means that the efficiency of the antenna will be affected more by conductivity and agrees with the high read ranges observed for users 5 and 7 who had the lowest tissue conductivity in the population. On the other hand, variation in permittivity led to a small shift in frequency. Fig. 10 shows decreasing frequency with increasing permittivity and a minimal change in return loss.

## 4 Conclusion

The influence of variation in body tissue composition on skin mounted UHF RFID tags has been shown in this Letter. A strong correlation between tissue permittivity and conductivity with read range has been demonstrated and correlations between BMI and read range have been shown for different body types with reasonable trends found for women and men with LM. The BMI relationship to tag performance can have use in developing simple models to allow users of wireless skin mounted sensors to assess the likely effect on performance for different body types. However, separate models are likely to be required for individuals with high muscle and low fat content. The conductivity of the skin tissue is seen to have a significant effect on tattoo tag efficiency and read range, while the permittivity moderately affects the tuned frequency.

## 5 Funding and Declaration of Interests

This work and Mr Oyeka’s studentship are funded by the UK Engineering and Physical Science Research Council (EPSRC, EP/J000825/1). Conflict of interest: None declared.
